# Treatment for Spontaneous Intracranial Dissecting Aneurysms in Childhood: A Retrospective Study of 26 Cases

**DOI:** 10.3389/fneur.2016.00224

**Published:** 2016-12-06

**Authors:** Yi-Sen Zhang, Shuo Wang, Yang Wang, Zhong-Bin Tian, Jian Liu, Kun Wang, Jun-Fan Chen, Xin-Jian Yang

**Affiliations:** ^1^Department of Interventional Neuroradiology, Beijing Neurosurgical Institute, Beijing Tiantan Hospital, Capital Medical University, Beijing, China; ^2^Department of Neurosurgery, Beijing Tiantan Hospital, Capital Medical University, Beijing, China; ^3^Department of Neurosurgery, The First Affiliated Hospital, Nanchang University, Nanchang, China

**Keywords:** intracranial aneurysm, pediatrics, dissection, intervention, treatment

## Abstract

**Objective:**

This study aimed to assess the clinicoradiological features and treatment outcomes of intracranial dissecting aneurysms (IDAs) in childhood.

**Methods:**

We conducted a retrospective study of pediatric patients who were treated for spontaneous IDAs in our institute between January 2010 and December 2015. The clinical presentation, aneurysm characteristics, treatment modality, and outcome were studied.

**Results:**

We studied 26 pediatric patients (mean age, 13.4 years; range, 4–18 years) with 31 IDAs who comprised 6.9% of all IDA patients treated during the same period. Seventeen (65.4%) patients were males, and nine (34.6%) were females. The incidence of large (≥10 mm in size) or giant aneurysms (≥25 mm in size) was 65.5%. Twenty-one (80.8%) patients underwent endovascular or surgical treatment and five (19.2%) received conservative treatment. Perioperative complications occurred in three patients, in whom two eventually recovered completely with a Glasgow Outcome Scale (GOS) score of 5 and one partially recovered with a GOS score 4. Overall, 25 (96.2%) patients had a favorable outcome and one (3.8%) had an unfavorable outcome at a mean follow-up of 22.8 months (range, 6–60 months).

**Conclusion:**

Pediatric IDAs are rare. In this series, endovascular management was a relatively safe and effective method of treatment for pediatric IDAs. However, continued follow-up is required because of the possibility of aneurysm recurrence and *de novo* aneurysm formation after treatment.

## Introduction

Intracranial aneurysms are rare in childhood, and intracranial dissecting aneurysms (IDAs) are even rarer (1, 2). The pediatric patients with an intracranial aneurysm manifested themselves clinically in various manners with hemorrhage, mass effect, or ischemia. They may also be found incidentally (3). Aneurysms in children show different features of etiology, sexual prevalence, location, and morphology compared with adults (3). Giant aneurysms are relatively common among pediatric patients with intracranial aneurysms (3). There have been few reports on children with IDAs (4, 5). This study aimed to analyze the clinical presentation, aneurysm characteristics, and treatment outcome of IDAs in patients aged 18 years or younger at our center over the last 6 years.

## Materials and Methods

This retrospective study was approved by our institutional review board. Written research consent was obtained from all study participants.

### Selection of Patients and the Population

Between January 2010 and December 2015, a total of 3,183 patients were hospitalized at our institution for treatment of a cerebral aneurysm. We retrospectively conducted a medical chart and imaging review of a prospectively collected neurovascular database to identify all patients with IDAs. Computed tomographic angiography (CTA), digital subtraction angiography (DSA), magnetic resonance angiography (MRA), and magnetic resonance imaging (MRI) were used to diagnose the IDAs. The diagnosis of IDA was made when CTA, MRA, or DSA showed fusiform dilation of vessels or pearl-and-string sign. IDA was also diagnosed when MRI showed intimal flaps, double-lumen sign, or intramural hematoma. Patients who were lost to follow-up or patients with acute traumatic aneurysms were excluded. Finally, a total of 59 pediatric intracranial aneurysms (31 dissected and 28 saccular) were identified. Twenty-six pediatric (6.9%) patients with 31 IDAs and 351 adult patients (93.1%) with 377 IDAs were included in this study. We collected information on patients’ demographics (age, sex, and clinical history), size, and location of IDAs, endovascular treatment selected, treatment complications, and angiographic and clinical follow-up outcomes.

### Institution’s General Treatment Approach

The optimum treatment for patients with IDAs is unknown, and there is little treatment experience in children with IDAs (1, 6). At our institute, patients with ruptured IDAs or with an obvious mass effect were treated as soon as possible if they could tolerate the operation. However, patients with unruptured IDAs and no obvious mass effect were initially evaluated with MRI/MRA and treated conservatively. Among these patients, those with ischemic symptoms were treated with antiplatelet agents, those with headaches and other atypical symptoms were treated symptomatically, and those without an obvious symptom and detected incidentally received conservative observation. If initial symptoms frequently recurred, or if the dissection site was shown to be continuously enlarged on follow-up MRI or MRA, angiography was performed to further evaluate the lesion. Endovascular or surgical treatment was then scheduled after risk–benefit evaluation for selected patients. Generally, endovascular treatment is the first choice of operation for pediatric patients with IDAs at our institute. However, for patients with a large acute intracranial hematoma, or for patients who had an IDA in superficial region of the brain and whose aneurysm was hard to be navigated for the microcatheter, microsurgical clips with or without artery bypass is an alternative. Among the endovascular treatment modalities, the first choice of operation is internal trapping, while coiling embolization and stent-assisted coiling are viable alternatives at our institute.

### Endovascular Procedures

**Figure 1:**
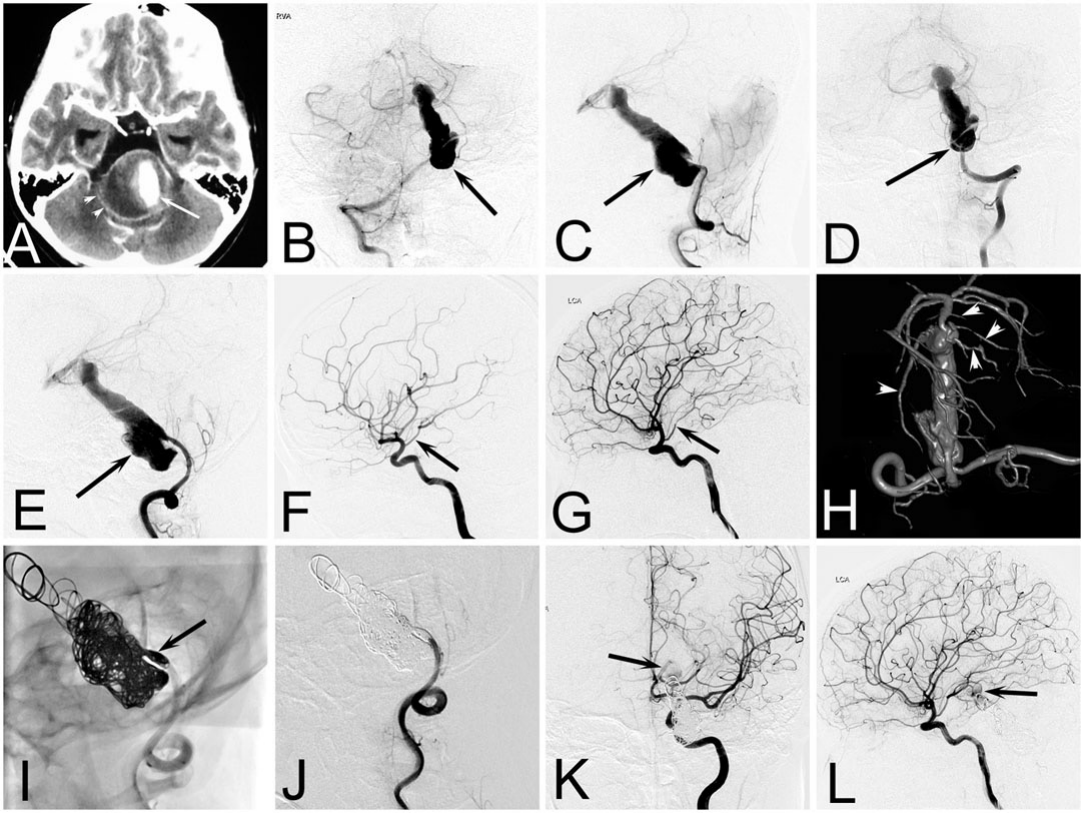
**Case of a 12-year-old girl who presented with acute headache complicated with diplopia and neck rigidity**. **(A)** Two-dimensional computed tomographic angiography showing a giant aneurysm of basilar artery with serious mass effect. **(B–E)** Right vertebral angiogram **(B,C)** and left vertebral angiogram **(D,E)** showing a dolichoectatic dissecting aneurysms of middle and lower segment of the basilar artery. **(F,G)** Right internal carotid artery (ICA) **(F)** and left ICA **(G)** angiogram showing the patency of bilateral posterior communicating artery (arrow). **(H)** A 3D reconstruction of a left vertebral angiogram showing the branches of upper segment of basilar artery (arrow heads). **(I,J)** The mask image **(I)** and immediate left vertebral angiogram **(J)** showing the lower part of the aneurysm as well as the distal end of left vertebral were completely embolized. **(K,L)** Immediate left ICA angiogram showing the left posterior cerebral arteries as well as upper segment of basilar artery (arrow) appearing *via* the left posterior communicating artery.

Endovascular procedures were performed under general anesthesia. When patients were scheduled for internal trapping, the balloon occlusion test was performed first in selected patients to evaluate compensatory blood supply. If the blood supply was sufficient, then various platinum coils were used to trap the aneurysm, as well as the parent artery proximal to the aneurysmal dilation (Figure 1). When patients were scheduled for reconstructive treatment, they received intravenous heparin during the interventional procedure and antiplatelet therapy before and after the intervention. For patients with unruptured IDAs, a daily dose of 1 mg/kg clopidogrel and 100 mg aspirin were administered for 3–5 days before treatment; for the only one pediatric patient with ruptured aneurysms and received stent-assisted coiling (case 7, 18 years old), he was loaded with 300 mg clopidogrel and 300 mg aspirin 4 h before treatment. Dual antiplatelet agents (1 mg/kg clopidogrel and 100 mg aspirin) were given orally once daily for 3 months after the procedure. Following this, 100 mg aspirin was continued for the next 3 months. Platinum coils were used to embolize the aneurysms, and stents were used to reconstruct the entire dissected segment.

### Materials

Various types of detachable coils were used in endovascular treatment. Self-expanding neurovascular stents, such as Enterprise (Cordis Neurovascular, Miami, FL, USA) and Solitaire AB (Ev3, Irvine, CA, USA) stents, were used to reconstruct the dissected artery.

### Follow-up

Follow-up angiographic examinations were performed with conventional MRA, CTA, or DSA at 3–6 months. If the aneurysms were shown to be completely occluded or stable, patients were then annually followed up with MRA. The patients’ outcomes were determined using the Glasgow Outcome Scale (GOS) score through a neurological examination at follow-up visits or by assessing the patient’s neurological status during a telephone interview. The scores at the latest follow-up were used in our analysis. A GOS score of 5 or 4 was taken to be a favorable outcome, and scores of 3, 2, or 1 were considered unfavorable (6).

## Results

### Clinical Profile

**Figure 2:**
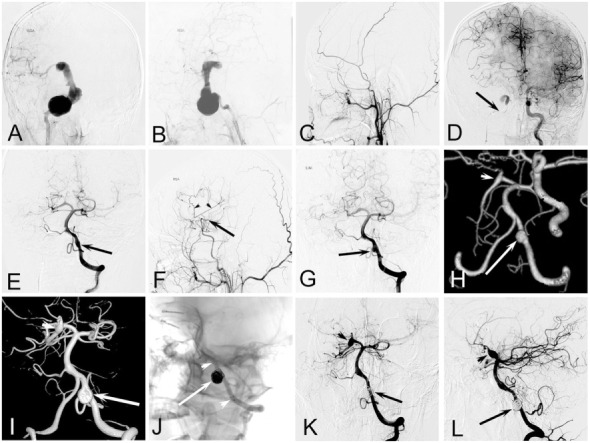
**Case of an 11-year-old boy who presented with continuing headache complicated with nausea and vomiting**. **(A,B)** Right internal carotid artery (ICA) angiogram showing a dissecting aneurysm at C4–C7. **(C)** Right external carotid artery angiogram after balloon occlusion of right ICA showing there was no blood supply from external carotid artery to brain. **(D)** Left ICA angiogram after balloon occlusion of right ICA showing the patency of the anterior communicating artery and the adequate blood supply of right anterior cerebral artery, but right middle cerebral artery (MCA) was absent. **(E)** Left vertebral angiogram after balloon occlusion of right ICA showing the patency of bilateral posterior communicating artery, a fusiform dilation of left vertebral artery (VA) (arrow). **(F)** Right external carotid artery angiogram 6 months after clipping of the aneurysm combined with superficial temporal artery–MCA bypass showing the patency of vascular anastomosis. **(G)** Left vertebral angiogram 6 months after operation showing the enlargement of the fusiform dilation of left VA (arrow). **(H)** Computed tomographic angiography 2 years after operation showing the further enlargement of the VA aneurysm (arrow) and a *de novo* fusiform aneurysm at right posterior cerebral artery (PCA) (arrow head). **(I)** A 3D reconstruction of a left vertebral angiogram 3 years after operation showing the further enlargement of the aneurysms at VA (arrow) and right PCA (arrow head). **(J)** The mask image showing the aneurysm at VA was treated by stent-assisted coiling. **(K,L)** Left vertebral angiogram 8 months after the intervention shows perfect reconstruction of the dissecting artery, and the aneurysm at right PCA was relatively stable.

The mean age of the 26 pediatric patients was 13.4 years (range, 4–18 years). The patients comprised 17 (65.4%) males and 9 (34.6%) females. Two patients had remote head trauma, though the causal relationship between trauma and IDAs was hard to identify. No related family history of intracranial aneurysms or genetic concomitant comorbidities was found in the 26 pediatric patients. Table 1 shows the patients’ main clinical presentation. Eight (30.8%) patients mainly presented with cerebral ischemia, eight (30.8%) with a mass effect, five (19.2%) with subarachnoid hemorrhage, three (11.5%) with headaches, and two (7.7%) with incidental lesions. The five patients with ruptured aneurysms all had a Hunt and Hess Grade of 1. Two patients had multi-aneurysms (cases 5 and 21). Case 5 (Figure 2), who had a giant IDA at C4–C7 of the right internal carotid artery (ICA) at onset, had two *de novo* IDAs at the left vertebral artery (VA) and right posterior cerebral artery (PCA) during follow-up. Case 22, who had encephalitis 1 month ago, had four mycotic aneurysms at the right ICA bifurcation, M2 segment of the right middle cerebral artery (MCA), right vertebral–basilar junction, and right posterior inferior cerebellar artery (PICA).

**Table 1:** **Details of 26 pediatric patients with intracranial dissecting aneurysms**.

Case No.	Age/sex	Main symptom	Site	Size (mm)	Length (mm)	Treatment modality	Perioperative complications	Radiological follow-up	GOS	Period (months)
**Patients who were operated**										
1	17/M	Mass effect	Basilar trunk	10	15	SAC	–	Occluded	4	36
2	18/M	Ischemia	Basilar trunk	5	5	SAC	–	Occluded	5	42
3	15/M	Mass effect	C6	10	15	Internal trapping	–	Occluded	5	27
4	11/M	SAH	P2	15	10	Internal trapping	–	Occluded	5	24
5	11/M	Mass effect	C4–C7	20	70	Clip + bypass	–	Occluded	5	34
			V4	10	12	SAC	–	Occluded		
			P2	3	10	Conservative	–	Stable		
6	15/M	SAH	P1	25	10	Coiling	–	Recurrent	5	21
7	18/F	SAH	Basilar trunk	9	9	SAC	–	Occluded	5	17
8	18/F	Mass effect	Basilar trunk	13	40	Internal trapping	–	NA	4	6
9	17/F	Headache	Proximal PICA	15	12	Aneurysmectomy	Wallenberg syndrome	Occluded	3	19
10	8/M	Ischemia	P2	55	30	Internal trapping	Thalamic infarction	Occluded	5	16
11	4/M	Headache	V4	17	23	Internal trapping	–	Occluded	5	22
12	7/F	Ischemia	M2	12	6	Internal trapping	–	Occluded	5	15
13	8/M	SAH	V4	30	7	Internal trapping	–	Occluded	5	13
14	16/F	Ischemia	Basilar trunk	15	12	Internal trapping	–	Occluded	5	18
15	6/F	Ischemia	P2	10	5	Internal trapping	–	Occluded	5	15
16	18/M	Ischemia	Basilar trunk	5	8	SAC	–	Recurrent	5	13
17	18/M	Ischemia	P2	5	8	Internal trapping	Thalamic infarction	Occluded	4	12
18	18/M	Mass effect	C4	25	20	Internal trapping	–	Occluded	4	12
19	6/F	Mass effect	V4	40	15	Internal trapping	–	Occluded	4	6
20	11/M	Mass effect	Basilar trunk	42	19	Internal trapping	–	Recurrent	4	6
21	12/F	Mass effect	Basilar trunk	30	55	Internal trapping	–	NA	4	6
**Patients not operated**										
22	13/M	Asymptom	C6–C7	13	20	Conservative	–	Stable	5	55
			M2	4	10		–	Stable		
			Basilar trunk	5	15		–	Stable		
			Proximal PICA	3	8		–	Thrombosed		
23	15/M	Ischemia	P1–P3	4	30	Conservative	–	Stable	5	60
24	18/M	Asymptom	M1	7	10	Conservative	–	Stable	5	48
25	18/F	Headache	VA–BA junction	12	28	Conservative	–	Thrombosed	4	24
26	12/M	SAH	Basilar trunk	7	10	Conservative	–	Thrombosed	5	26

*GOS, Glasgow Outcome Scale score at the latest follow-up; SAC, stent-assisted coiling; SAH, subarachnoid hemorrhage; PICA, posterior inferior cerebellar artery; VA–BA, vertebral artery–basilar artery; NA, not available*.

### Aneurysm Characteristics

The distribution of the 31 IDAs in pediatric patients based on location is shown in Table 2. There were 7 (22.6%) aneurysms in the anterior circulation and 24 (77.4%) in the posterior circulation. The most common location of aneurysms was the basilar artery (*n* = 10, 32.2%), followed by the PCA (*n* = 7, 22.6%), ICA (*n* = 4, 12.9%), VA (*n* = 4, 12.9%), MCA (*n* = 3, 9.7%), PICA (*n* = 2, 6.5%), and vertebral–basilar junction (*n* = 1, 3.2%). Of the 32 pediatric IDAs, the mean length of the dissecting artery was 17.6 mm (range, 5–70 mm), and the mean widest diameter of aneurysmal dilation was 15.4 mm (range, 3–55 mm). According to the widest diameter of aneurysmal dilation, there were 20 (64.5%) large (≥10 mm) or giant aneurysms (≥25 mm).

**Table 2:** **Aneurysm location in pediatric patients**.

Location	No. of aneurysms (%)
Posterior circulation	24 (77.4)
Basilar artery	10 (32.2)
Vertebral artery	4 (12.9)
Vertebral–basilar junction	1 (3.2)
Posterior cerebral artery	7 (22.6)
Posterior inferior cerebellar artery	2 (6.5)
Anterior circulation	7 (22.6)
Internal carotid artery	4 (12.9)
Middle cerebral artery	3 (9.7)
Total	31 (100)

### Treatment Modality and Outcome

Among the 31 aneurysms, endovascular treatment was performed in 20 (64.5%), surgical treatment in 2 (6.5%), and conservative treatment in 9 (29.0%). Clinical follow-up was available in all patients with a mean duration of 22.8 months (range, 6–60 months), and imaging follow-up was available in 24 (92.3%) patients. At the last follow-up, 25 (96.2%) patients had a favorable outcome with a GOS score of 4–5 and 1 (3.8%) had an unfavorable outcome with a GOS score of 3.

Of the 20 IDAs that were treated endovascularly, 14 were treated with internal trapping, 5 with stent(s)-assisted coiling, and 1 with coil embolization. None of the patients had intraoperative complications. Two patients (cases 10 and 17) had postoperative ischemic complications but totally recovered during the follow-up of 6 months. Of the 20 aneurysms, radiographic follow-up was available in 18 (90.0%); 15 (83.3%) of these showed stable occlusion and 3 (16.7%) showed recurrence and needed a second treatment (cases 6, 16, and 20). Case 6, who had a ruptured IDA at the P1 segment and received coil embolization for first treatment, had recurrence of the aneurysm after another 6 months. This patient received internal trapping for the second treatment and had complete occlusion at a 6-month follow-up. Case 16, who had an IDA in the upper part of the basilar artery, was treated with Enterprise stent-assisted coiling for the first treatment and had recurrence of the aneurysm 5 months later. For further treatment, this patient was treated by overlapping with another Enterprise stent and coil embolization, and no recurrence was observed in the radiographic follow-up after 6 months. Case 20, who had an IDA at the V4 segment and received internal trapping for the first treatment, had antegrade recurrence of the aneurysm 3 months later. Further treatment was scheduled, and she was waiting for a second endovascular treatment.

Of the two patients who had aneurysms treated surgically, there were no intraoperative complications, but one patient had postoperative complications. One of the patients (case 9), who had an aneurysm at the proximal segment of the PICA and received aneurysmectomy, presented with Wallenberg syndrome after the operation. She received mechanical ventilation treatment in an intensive care unit for 1 month and slowly recovered. After 1 year, she partially recovered with a GOS score of 3. These two patients did not have any recurrence in the radiographic follow-up.

Of the nine aneurysms that were not embolized or clipped, two had spontaneous aneurysm thrombosis before endovascular treatment, one showed spontaneous thrombosis during follow-up, and the other six aneurysms were stable during follow-up.

## Discussion

Intracranial dissecting aneurysms in childhood are rare, and endovascular approaches have become the major operative modality for IDAs (3). However, little is known about the mid-term or long-term results of endovascular treatment of IDAs. In this study, we examined the clinical outcomes of 26 consecutive pediatric patients with IDAs over the last 6 years.

### Incidence of IDAs

Intracranial dissecting aneurysms are much less frequent in children than in adults (1). In our study, only 6.6% of the patients were 18 years or younger among all the patients with IDAs. A similar proportion was found in other studies (7, 8). In a North American single-center series of 263 consecutive patients with cervicocephalic dissections, 18 (7%) occurred in children, of which 11 (4.2%) were intracranial (9). Our study showed that males accounted for the majority children with IDAs (68%), and large or giant aneurysms were relatively common, which is consistent with other reports (10–12). Further study was needed in multicenter with a large sample to define specific epidemiological data of IDAs in pediatric patients.

### Site of Aneurysms

Pediatric IDAs are more likely to occur in the posterior circulation (1, 11, 13), as found in this case series. According to Saraf et al. (3) and Lasjaunias et al. (14), dissecting aneurysms in pediatric patients are more likely to be located in the PCA, the supraclinoid ICA, and the MCA. However, in our study, the most common location of aneurysms was the BA. Taking into account that our study only included 26 single-center patients, the high incidence of BA aneurysms might be due to a higher referral of complex cases to our specialized center.

### Endovascular Treatment of IDAs

During the past 10–20 years, there has been a major shift from microsurgical treatment toward endovascular management because of better results, and lower rates of morbidity and mortality in patients with IDAs (15). The outcome following endovascular treatment was favorable in all patients in this study. A similar experience had been previously reported in pediatric patients (15).

Internal trapping was our first choice of treatment, which was based on the following two points. First, most IDAs in children are large or giant aneurysms, and the recurrence rate of reconstructive treatment is high (16). Second, pediatric patients can tolerate deconstructive treatment better than adults because of a greater functional brain capacity and a better compensatory blood supply (10, 17). Our study showed that the efficacy of internal trapping was satisfactory and only one patient had recurrence of an aneurysm. Even for IDAs that were located on the BA or the terminal branch of the artery, internal trapping could still be considered. Four cases (cases 8, 14, 20, and 21), who had IDAs involved in the basilar trunk, received internal trapping and had favorable outcomes (e.g., case 21; Figure 1). Two cases (cases 10 and 17), who had IDAs in the P2 segment and reconstructive treatment limited to the small diameter of the parent artery, finally also received internal trapping. Although transient unilateral limb asthenia occurred after the interventional procedure, the two patients recovered soon after conservative treatment, and there were no operation-related sequela after 6 months.

Endovascular reconstructive treatment is an alternative method when internal trapping is limited. This modality is controversial because of its relatively higher recurrence rate during follow-up (16). However, with major progress in endovascular techniques, such as flow-diverting stents, the recurrence rate in adult patients with IDAs after endovascular reconstructive treatment has significantly declined in recent years (18). In China, the flow-diverting stent is only used in patients aged 22 years or older according to the Food and Drug Administration, and we do not use this instrument in pediatric patients.

### Microsurgical Treatment of IDAs

Microsurgical treatment has not been used as frequently in our institute in recent years because of a high surgical risk. One (case 9) of the two patients who received craniotomy had postoperative complications in our study. Agid et al. (19) and Lasjaunias et al. (20) compared surgical and endovascular treatments in children with cerebral aneurysms, and they concluded that endovascular treatment provides a better clinical outcome. However, microsurgical treatment still plays an irreplaceable role in particular patients. For patients with a serious mass effect or large intracranial hematoma, or for those whose endovascular treatment is limited, microsurgical clips with or without artery bypass is an alternative method (1). One patient (case 5; Figure 2) had an IDA at the C4–C7 segment of the right ICA, and compensatory blood supply was insufficient after ICA occlusion. This patient had clipping of the aneurysm combined with superficial temporal artery–MCA bypass and had a favorable outcome.

### Conservative Treatment of IDAs

Conservative treatment has gradually been accepted as a common management for unruptured pediatric aneurysms (1). In earlier series, patients with IDAs without SAH were often offered surgical or endovascular treatment because of concern that the dissecting aneurysm would rupture (1, 21). However, in recent years, most adult patients with intracranial artery dissection without SAH have been treated medically and offered acute stroke treatment and long-term prevention of ischemic stroke (1, 22). In our clinical practice, conservative treatment is the main management for pediatric patients with unruptured IDAs in outpatient clinics. This is because many unruptured IDAs have a relatively benign cause and can even repair themselves (23). In our case series, three aneurysms had spontaneous thrombosis, which is an uncommon event in the adult population (13). Koroknay-Pal et al. (24) and Liang et al. (25) also reported the same phenomenon. A mural hematoma appears to be the most important promoting factor for spontaneous thrombosis and healing of aneurysms (4).

### Follow-up of IDAs

Regular radiographic follow-up is important in pediatric patients with IDAs because of the possibility of recurrence after treatment and a high rate of *de novo* aneurysm formation in children (14). In this study, one case (case 5) had two *de novo* IDAs within 2 years after the first IDA was clipped. Kakarla found that most *de novo* aneurysms form within 3 years after the initial debut of disease (26). We also agree with this author’s recommendation that life-long follow-up with MRI is mandatory, and we suggest imaging for screening purposes at 3-year intervals (26).

### Limitations

There are some limitations to this study. These limitations include the retrospective design, patient selection bias, and a limited number of cases in a single institution.

## Conclusion

Pediatric IDAs are rare. Among pediatric patients with IDAs, the majority are male, and large or giant aneurysms are relatively common. In this series, endovascular management was a relatively safe and effective method of treatment for pediatric IDAs. However, continued follow-up is required because of the possibility of aneurysm recurrence and *de novo* aneurysm formation after treatment.

## Author Contributions

Y-SZ drafted the manuscript. SW, YW, and X-JY performed the operations in this study. Z-BT, JL, KW, and J-FC performed the data collection and data analysis. X-JY participated in the design of this study and helped to check the manuscript.

## Conflict of Interest Statement

The authors declare that the research was conducted in the absence of any commercial or financial relationships that could be construed as a potential conflict of interest.
